# Design and Experimentation of a Machine Vision-Based Cucumber Quality Grader

**DOI:** 10.3390/foods13040606

**Published:** 2024-02-16

**Authors:** Fanghong Liu, Yanqi Zhang, Chengtao Du, Xu Ren, Bo Huang, Xiujuan Chai

**Affiliations:** 1 State Key Laboratory of Robotics and System, Harbin Institute of Technology, Harbin 150001, China; 2 Agricultural Information Institute, Chinese Academy of Agricultural Sciences, Beijing 100081, China

**Keywords:** quality grader, cucumber grading, deep learning, mass prediction

## Abstract

The North China type cucumber, characterized by its dense spines and top flowers, is susceptible to damage during the grading process, affecting its market value. Moreover, traditional manual grading methods are time-consuming and labor-intensive. To address these issues, this paper proposes a cucumber quality grader based on machine vision and deep learning. In the electromechanical aspect, a novel fixed tray type grading mechanism is designed to prevent damage to the vulnerable North China type cucumbers during the grading process. In the vision grading algorithm, a new convolutional neural network is introduced named MassNet, capable of predicting cucumber mass using only a top-view image. After obtaining the cucumber mass prediction, mass grading is achieved. Experimental validation includes assessing the electromechanical performance of the grader, comparing MassNet with different models in predicting cucumber mass, and evaluating the online grading performance of the integrated algorithm. Experimental results indicate that the designed cucumber quality grader achieves a maximum capacity of 2.3 t/hr. In comparison with AlexNet, MobileNet, and ResNet, MassNet demonstrates superior cucumber mass prediction, with a MAPE of 3.9% and RMSE of 6.7 g. In online mass grading experiments, the grading efficiency of the cucumber quality grader reaches 93%.

## 1. Introduction

**Figure 1:**
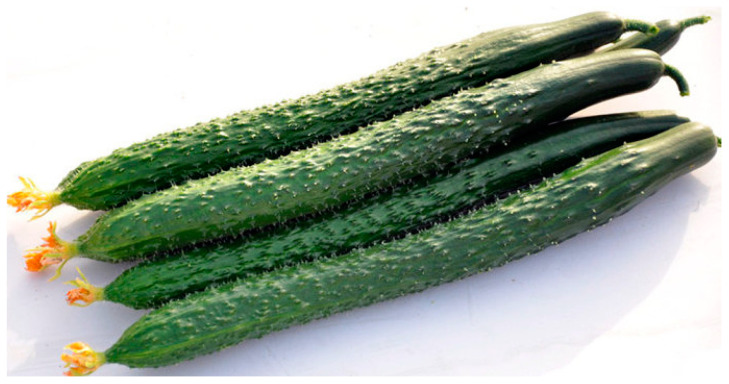
North China type Cucumbers.

The cucumber (*Cucumis sativus* L.) is a popular and extensively cultivated vegetable with a high content of vitamins, fiber, and mineral elements [1]. China is the world’s largest producer of cucumbers [2], primarily cultivating the North China type, as shown in Figure 1. Unlike cucumbers produced in other regions [3], the main characteristics of North China type cucumbers include dense spines and vibrant flowers. Grading cucumbers can enhance their commercial value and farmers’ profits [4,5]. However, in China, cucumber grading is mainly reliant on manual labor due to the vulnerability of spines and flowers on North China type cucumbers to damage. Damage to the dense spines and flowers accelerates moisture loss, which is perceived by consumers as a lack of freshness. Therefore, there is an urgent need to develop an automatic, non-destructive cucumber grading technology and corresponding equipment. With the widespread application of machine vision and mechatronics technology in agricultural production [6,7,8,9], an effective approach is provided for the design of a cucumber grading machine.

Machine vision is a technology that mimics the human visual system, having its origins dating back to the 1960s [10]. With the rapid advancement of semiconductor and computer technologies in the 1990s, machine vision experienced significant progress in various fields. The working principle of machine vision involves using various imaging methods to acquire optical images of the object under detection. These images are processed and analyzed by a computer to extract the required information [11]. In the food industry, the main applications of machine vision include quality detection, sorting, and grading [12,13]. The quality detection and grading of agricultural products are important stages in the pre-processing of the food industry. Utilizing machine vision for quality analysis of agricultural products enables rapid and accurate assessment and grading. This not only reduces the manual labor intensity but also ensures consistency in product quality. Haiyan Cen et al. [14] utilized hyperspectral images and PCA methods to detect cucumbers with internal hollow defects in the food processing line, achieving an accuracy of 95.1%. This method addresses the issue of cucumber bloating and rupture during food processing due to internal hollowness. I. R. Doni-González et al. [15] utilized machine vision and a fourfold cross-validation Neural Network Classifier to achieve detection and grading of the internal fibrous tissue of carrots, with an accuracy of 86.4%. This method was applied in the processing of diced carrots for infant consumption, effectively preventing the risk of choking for infants caused by carrots with undesirable fibrous tissue. Chengqian Jin et al. [16] processed 32-bit RGB (Red, Green, Blue) cucumber images, applied an approximate circular method to fit the cucumber’s central axis, established a curve equation, obtained the cucumber’s curvature, and utilized curvature for grading, achieving an accuracy of 99.3%. These studies have achieved good results in quality detection and grading of food using a combination of machine vision and traditional machine learning. But there are drawbacks, such as sensitivity to environmental lighting and complex scenes, as well as a lack of generalization. The emergence of deep learning methods effectively addresses these shortcomings. The integration of machine vision and deep learning technologies enables better handling of the demands for food quality detection and grading in diverse and complex scenarios. Deep learning can fully leverage image data, extract implicit features, and enable end-to-end processing. In recent years, it has gained popularity in grading various rod-shaped fruits and vegetables. Jingjun Cao et al. [17] used deep learning to achieve defect grading of zizania with an accuracy of 95.6% Furthermore, this method also performs well in apple grading, demonstrating excellent generalization. Hongfei Zhu et al. [18] established a carrot shape grading model based on transfer learning technology, achieving classification of normal/abnormal shapes with an accuracy of 98%. Ahmad Jahanbakhshi et al. [19] improved the pooling function in the CNN model, and the enhanced deep learning model can classify regular and irregular carrot shapes with an accuracy of 99.43%. These studies used deep learning and machine vision in the process of food quality detection and grading, which fully utilizes the information from images, enhances generalization, and maintains stable accuracy when facing complex scenarios. This ensures the quality and safety of food entering the market or food processing stages. However, in the research on cucumber quality detection and grading, few researchers have adopted machine vision and deep learning methods.

In the research on grading systems and equipment for rod-shaped fruits and vegetables, Yee-Siang Gan et al. [20] combined deep learning to design an online cucumber grading system. After visual detection, cucumbers were graded by blocking the rolling cucumbers with a baffle. Limiao Deng et al. [21] designed an online carrot grading system, which used pneumatic components to blow visually detected carrots into the corresponding grading boxes. The V. K. Chong et al. [22] eggplant sorting machine employed trays carrying eggplants that, through flipping, caused the eggplants to fall into the corresponding grading chutes. In the study of grading systems and equipment for spherical fruits and vegetables, the majority also utilized lateral and vertical falling mechanisms for grading [23,24,25,26]. The grading mechanisms adopted by these grading systems and equipment achieved fruit and vegetable grading by creating a height difference. For cucumbers with vulnerable skin, a grading structure with height difference could increase mechanical damage, posing challenges to cucumber storage and sales. Therefore, there is an urgent need to develop equipment that provides protection for cucumbers with vulnerable skin during the grading process and enables quality grading.

In this context, the overall aim of the present research was the design and validation of a fresh cucumber quality grader based on machine vision and deep learning. To the best of our knowledge, the current study offers two signification new insights. Firstly, we have designed a novel height difference-free grading mechanism—fixed tray type, which aims to protect the North China type cucumber during the grading process. Meanwhile, we have provided a corresponding electrical control strategy to ensure grader capacity. Secondly, we have developed a new network architecture model—MassNet, which can accurately predict the mass of a cucumber solely based on its RGB top-view image. Finally, experiments have been conducted on the designed grader and algorithm: (1) Key electromechanical performance parameter of the grader capacity, (2) Comparison of the performance of AlexNet, MobileNet, ResNet, and MassNet in predicting cucumber mass, and (3) Online grading validation after integrating the algorithm into the grader.

## 2. Materials and Methods

### 2.1. Overview of the Cucumber Quality Grader Design and Experiments

**Figure 2:**
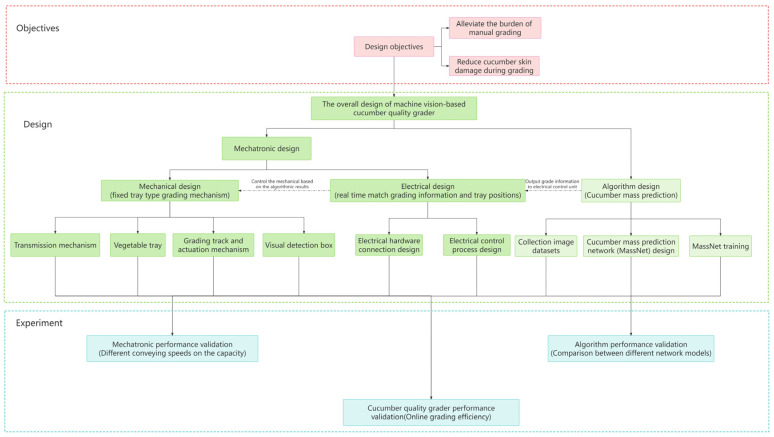
The cucumber quality grader design and experiments flow schematic.

The cucumber quality grader design and experiments flow schematic is shown in Figure 2. Starting from the goal of reducing damage to the skin of North China type cucumbers during grading and alleviating the burden of manual grading, we designed a cucumber quality grader based on machine vision. The overall design of the grader includes a mechatronic design and algorithm design. In the mechatronic design, a fixed tray grading mechanism and an electrical control strategy for grading information and tray positions were developed, achieving real-time cucumber grading and protection. In the algorithm design, cucumber mass prediction was accomplished through the collection of image datasets, the design of a cucumber mass prediction network (MassNet), and training. In experiments, mechatronic performance was validated through capacity experiments at different conveying speeds. Algorithm performance was verified by comparing different network models and MassNet. The efficiency of the cucumber quality grader was confirmed through online experiments.

### 2.2. Cucumber Quality Grader Mechatronic Design and Grade Principle

**Figure 3:**
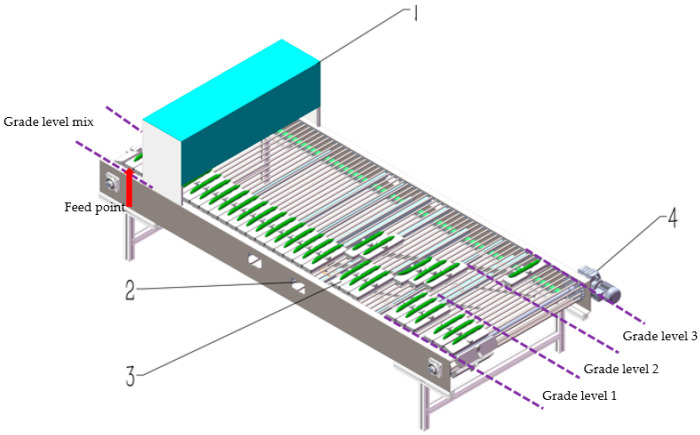
The overall structure and grading schematic of the cucumber quality grading machine. (1) vision detection box; (2) grading track with an actuation mechanism; (3) vegetable trays; (4) power unit.

The overall design of the cucumber quality grader includes both mechanical and electrical components. The main structure, as shown in Figure 3, comprises a vision detection box (for detecting and grading), a grading track with an actuation mechanism, vegetable trays (carriers for vegetable grading), and a power unit (providing power to the entire grading conveyor system). During the grading process, cucumbers with a grade level mix are placed on the tray at the feeding point and, driven by the chain wheels, move along the grading track, passing through the vision detection box to determine their grade information. After detection, the tray carrying the cucumbers continues along the grading track. Based on the quality grade information, with the assistance of the track-turnout actuation mechanism, it slides onto the corresponding grading track to achieve the grading process. Throughout the grading process, cucumbers remain on the tray without height difference, providing effective protection for the cucumbers.

#### 2.2.1. Mechanical Design

1. Transmission Mechanism

**Figure 4:**
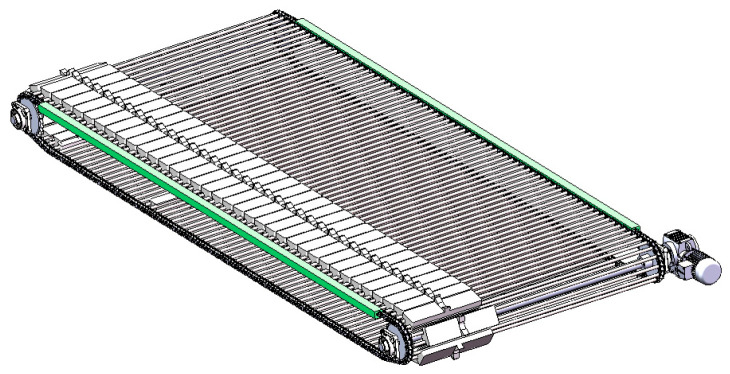
Transmission mechanism.

The transmission mechanism provides power for the entire grading and conveying system, using sprockets as illustrated in Figure 4. Both driving and driven sprockets have 27 teeth, with a transmission ratio of 1:1 and a sprocket center distance of 3500 mm. The overall operating condition involves low- to medium-speed operation under a light load. The roller chain is of type 16A, with a pitch of 25.4 mm. A stainless steel pipe with a diameter of 16 mm is installed on each chain link. Each vegetable tray is mounted on two adjacent stainless steel pipes, arranged in a single row. The entire power system utilizes a 750 W three-phase asynchronous AC motor, a 1:30 reducer, and a matching frequency converter to drive the sprockets’ rotation, with adjustable speeds ranging from 0.1 m/s to 0.5 m/s. During operation, the motor provides power, and the chain links engage with the driving sprocket, causing the vegetable trays mounted on the stainless steel pipes to move forward in the direction of the gear rotation.

2. Vegetable Tray

**Figure 5:**
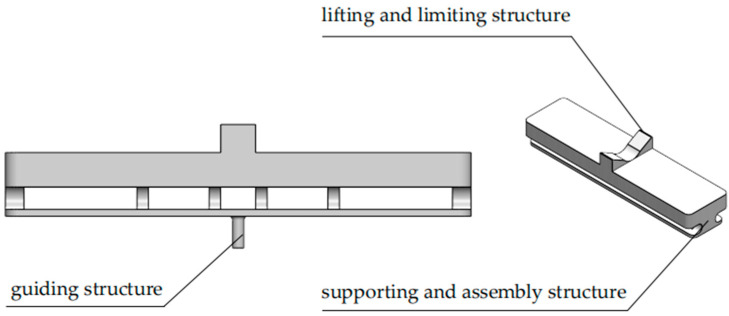
Vegetable tray.

As a conveyor for susceptible cucumbers, the vegetable tray provides protection during the grading process. The dimensions of the tray are 400 mm × 97 mm × 55 mm, comprising lifting and limiting structure, supporting and assembly structure, and guiding structure, as shown in Figure 5. The lifting and limiting structure, in direct contact with cucumbers, incorporates a U-shaped structure in the middle, limiting the movement of cucumbers in the conveying direction. This prevents them from rolling or falling out of the tray due to sudden stops, speed changes, and other conditions, ensuring the quality of image acquisition by the camera and preventing damage to the outer skin of cucumbers. The supporting and assembly structure utilizes a multi-ribbed support structure to enhance the overall tray strength, with a design featuring 19 mm through-holes for assembly. The bottom guiding structure consists of a cylinder with a diameter of 10 mm and a height of 16 mm. To reduce inertia and weight, the upper and middle sections of the entire tray are designed with a hollow structure with a wall thickness of 2 mm, while the bottom guiding structure is solid. The material for the entire tray is chosen to be high-performance fiber-reinforced nylon, considering the wear and significant impact that the bottom guiding structure may experience during the grading process.

3. Grading Track and Actuation Mechanism Design

In order to protect cucumbers during the grading process and ensure there is no significant height difference or rolling throughout the entire grading process, we designed a novel grading mechanism called the fixed tray type grading mechanism. The trays, designed as mentioned earlier, are mounted on stainless steel pipes of the conveying mechanism, with a single degree of freedom (only allowing Y-directional sliding). Guided by the grading track, the trays undergo grading through the actuation mechanism.

**Figure 6:**
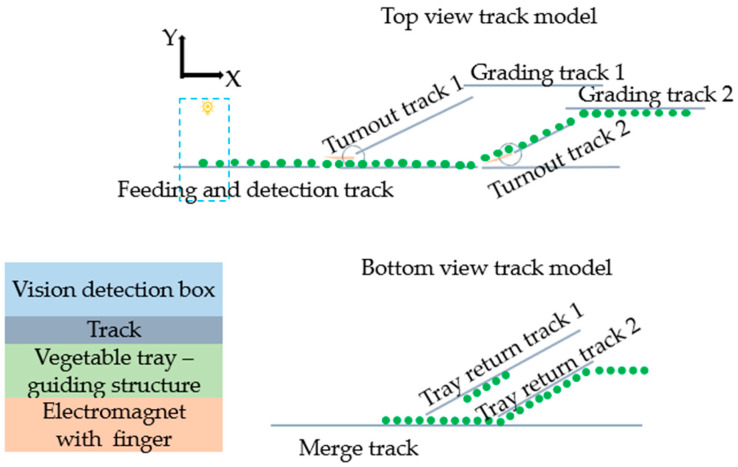
Grading track model.

The grading track and the actuation mechanism are crucial components for diverting the vegetable trays according to the intended path. The grading track is divided into the top track and the bottom track, as shown in Figure 6. The top track includes the feeding and detection track, turnout tracks, and grading tracks. The bottom track consists of the tray return tracks and the merging track. All tracks are mounted on the supporting pipes inside the sprocket conveyor, and except for the turnout, other tracks are implemented using U-shaped aluminum profiles.

The movement process of the vegetable tray on the track is as follows: when the vegetable tray is on the top track, it moves along the feeding track under the guidance of the guiding structure, completes visual judgment through the visual detection box, and approaches the turnout track. According to the visual grading result, the turnout performs the corresponding action, allowing the vegetable tray to slide onto the grading track, achieving the grading. After unloading the carried vegetables from the grading track, the vegetable tray continues to rotate to the bottom track, moving along the return track of each level until it reaches the merging track to reset the position of the vegetable tray. It then moves to the top track to load vegetables, repeating the above process to achieve continuous grading.

The turnout track plays a role in diverting and grading the vegetable tray in the entire grading process, requiring coordination with the turnout actuating mechanism to achieve tray diversion. The turnout actuating mechanism includes a finger and a driving component. The finger is used for the transition between the feeding and detection track and the turnout track, mounted on the driving component, with dimensions of 50 mm × 20 mm × 3 mm, made of aluminum alloy. The driving component can use pneumatic, hydraulic, mechanical, or electromagnetic driving. Considering the high-frequency and low-load characteristics of the grading machine, hydraulic and mechanical driving methods are excluded. Given the possibility of interference between the guiding rod of the vegetable tray and the turnout track during fast movement, the working time of the turnout actuating mechanism should not exceed 100 ms. Therefore, an electromagnet with high responsiveness and a rotation angle of 30° is chosen as the driving component for the turnout actuating mechanism.

4. Visual Detection Box

The visual detection box is designed to provide a stable and consistent environment for capturing fruit and vegetable images, reducing image noise and preprocessing, and facilitating subsequent visual grading analysis. The entire visual detection box includes a visual box, a camera, a light source, and a photoelectric sensor. The visual box is a stainless steel, sealed container providing installation positions for the camera, light source, and photoelectric sensor. Four sets of LED light strips are placed on the top of the box, and the interior is lined with black flexible paper to reduce specular reflection of light. The camera is positioned in the middle of the box, 40 cm away from the vegetable tray, allowing clear and complete capture of vegetables on the tray. The photoelectric sensor is placed on one side of the box, close to the side of the vegetable tray, at a distance of 2 cm from the tray.

#### 2.2.2. Electrical Design

1. Hardware

**Figure 7:**
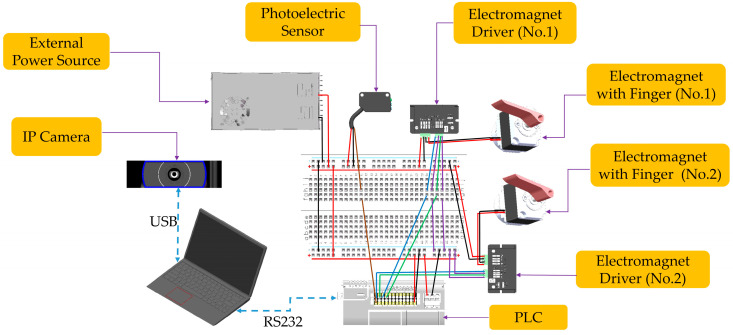
Hardware schematic.

The electrical hardware comprises components such as cameras, photoelectric sensors, electromagnets with drivers, a computer, the PLC (Programmable Logic Controller), and the power supply. The hardware schematic is illustrated in Figure 7. The signal lines from the photoelectric sensor and the driver of the electromagnet are connected to the corresponding interfaces on the PLC. Communication between the PLC and the computer is established using the RS232, while the camera is connected to the PC via USB. A 220 V to 24 VDC power supply is employed to provide power to the electromagnets and other electronic components.

2. Electrical control process

**Figure 8:**
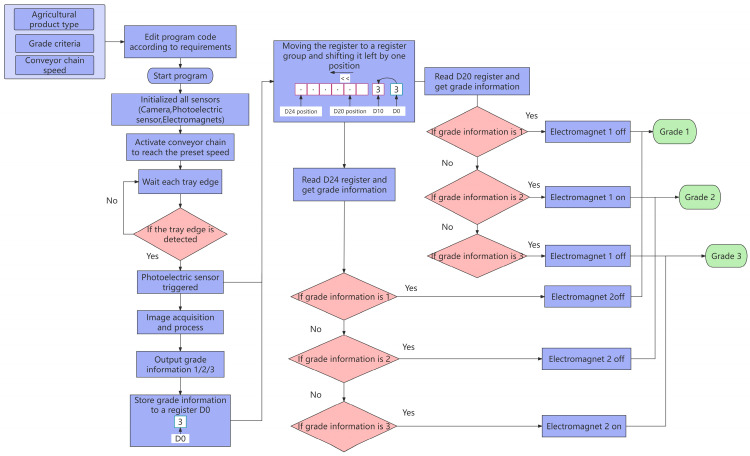
Electrical control process flow.

**Figure 9:**
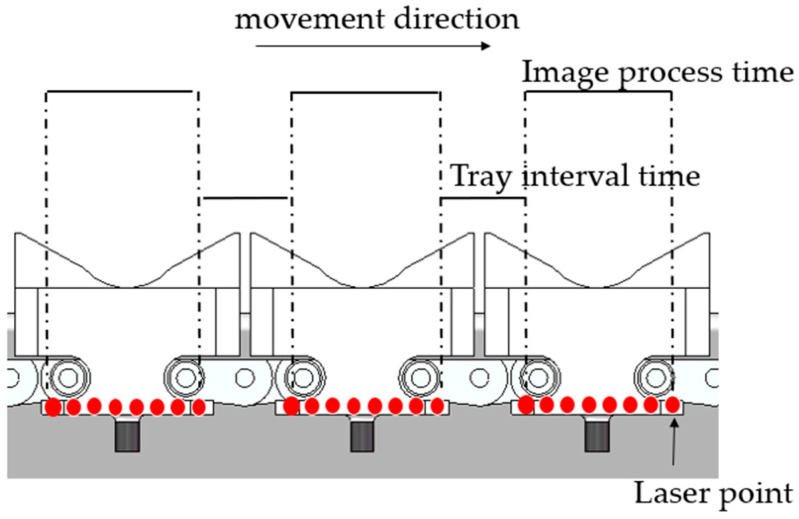
Image processing time and tray interval time.

The electrical control process of the entire system is depicted in Figure 8. Prior to initiation, project parameters, such as agricultural product type, grading criteria, and conveyor chain speed, are configured in the program. Upon startup, various sensors (camera, photoelectric sensors, electromagnetic coils) and a computerized system are initialized. Once the chain speed reaches the predetermined velocity, the system awaits the arrival of the vegetable tray. When the laser emitted by the photoelectric sensor illuminates the edge of the vegetable tray, the PLC notifies the upper computer, and the camera captures the current image of the vegetables on the tray for image processing. The output includes grade information 1, 2, and 3. When the laser emitted by the photoelectric sensor reaches the end of the edge of the vegetable tray, the corresponding grade information is stored in register D_0_, associating the grade information with the respective tray. The time from the first laser detection at the edge of the tray to the end of the tray represents the image processing time. The tray interval time is the response time triggered by the photoelectric sensor, as illustrated in Figure 9. Subsequently, for each trigger of the photoelectric sensor, the grading information stored in the register is copied to the tray position register group D_10_ and shifted left by one bit. The width of D_10_ is 14 bits. The photoelectric sensor detects each vegetable tray, causing all grading information in the D_10_ position register array to shift by one bit. This approach ensures real-time synchronization and tracking of grading information and tray positions.

The PLC consistently reads the X-th and X + 4-th bits of the D_10_ position register group, controlling the switches of electromagnets 1 and 2. If the grading information is 1, both electromagnets 1 and 2 are turned off, achieving the grading of the level 1 fruit and vegetable. For grading information 2, electromagnet 1 is activated, and electromagnet 2 is turned off, causing the vegetable tray to move along turnout track 1, achieving the level 2 grading. In the case of grading information 3, electromagnet 1 is turned off, and electromagnet 2 is activated, causing the vegetable tray to move along turnout track 2, achieving the level 3 grading.

### 2.3. Vision-Based Quality Grading Algorithm

Mass is one of the important indicators for cucumber quality grading. Most researchers use image procession or machine learning methods for mass prediction of agricultural products [27,28,29,30]. We have developed a Convolutional Neural Network (CNN)-based model for predicting the mass of North China type cucumbers, named MassNet. Unlike traditional machine learning approaches, MassNet eliminates the need for extracting complex explicit features, estimating the mass of cucumbers solely based on a top-view image. The cucumber mass predicted by this model is compared with the standard values outlined in the grading document, forming the basis for cucumber classification. Table 1 presents the mass grading requirements from the cucumber mass grading document (DB37/T564-2005) in Shandong Province [31].

#### 2.3.1. Collection Image Datasets

The collection of image data for North China type cucumbers was conducted at Hongji Agriculture Company (Zibo City, Shandong Province, China). The cucumbers gathered were freshly picked on the same day and retained the characteristics of top flowers with spines. The Logitech C920 webcam used for capturing images was identical to the camera model mounted on the intelligent grading machine. Cucumbers were placed on the designed vegetable tray for shooting, ensuring a disturbance-free background and minimizing image preprocessing. The cucumber dataset comprises three grades based on quality: Grade 1, Grade 2, and Grade 3. The dataset includes a total of 409 cucumbers, with 103, 159, and 147 cucumbers for Grade 1, Grade 2, and Grade 3, respectively. The cucumber weights range from 95 g to 248 g. Each cucumber was labeled and weighed using a calibrated electronic scale with a maximum capacity of 500 g and an accuracy of ± 1 g.

#### 2.3.2. Architecture of the Cucumber Mass Prediction Network

**Figure 10:**
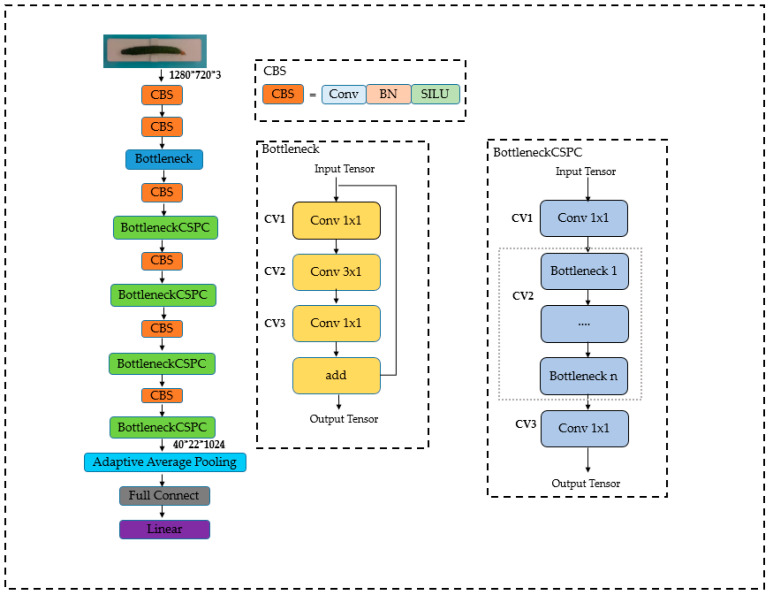
MassNet architecture.

MassNet adopts a structure alternating between convolutional and CSPC (Cross-Stage Partial Connections), which processes input images layer by layer, extracts crucial features, and ultimately achieves accurate prediction of cucumber quality. All convolutional operations undergo batch normalization and SILU. In the initial two convolutional operations, the network gradually reduces the spatial size of feature maps while retaining image details. The use of Bottleneck, while maintaining the channel number, employs stacked convolutional layers to learn complex feature representations, and residual connections ensure gradient persistence. The alternating use of Conv and BottleneckCSPC, based on residual connections, further introduces Cross-Stage Partial Connections [32] to progressively reduce the spatial dimension of feature maps, increase the channel number, extract higher-level abstract feature modules, enhance the network’s feature learning capability, and improve the model’s ability to extract image features of different scales and complexities. Feature maps are reduced to a fixed-size vector through adaptive average pooling. This design ensures that the model has robustness for variations in the scale of input images, allowing it to better adapt to cucumbers of different sizes. Finally, the dimension-reduced features are input into fully connected layers for quality prediction. The architecture of MassNet is shown in Figure 10.

#### 2.3.3. Training Settings

During the training process, we randomly split the dataset into a training set and a validation set with an 8:2 ratio. Since predicting the mass of cucumbers is a regression problem, we chose the Mean Squared Error loss function as the model’s loss function. In the realm of deep learning, the Adam optimizer is widely used due to its advantages, such as adaptive learning rates and momentum optimization, eliminating the need for manual adjustment of the learning rate. Therefore, in the training of MassNet, we opted for the Adam optimizer. Research indicates that Adam performs better in the application of quality grading for rod-shaped fruits and vegetables compared to SGD and Adadelta. Within the specified number of epochs, we save the model of the current epoch when the MSE value on the validation set is minimized, designating it as the quality prediction model. Table 2 is the detailed hyperparameter settings.

## 3. Results and Discussion

### 3.1. Effect of Different Conveying Speeds on the Capacity of Grader

#### 3.1.1. Experiment Setup

Capacity is the maximum measure that a machine can generate by executing the intended actions, serving as a crucial parameter reflecting the mechanical characteristics of the grader. The machine’s capacity is the ratio of the sample mass (t) to the time required for grading (hr) [33], as in Equation (1).

(1) $$\mathrm{capacity}=\frac{W_{F}}{T}$$

where *W_F_* represents the mass of fruits taken in t, and *T* represents the time taken for sorting and grading in hr.

In the experiment, a digital tachometer was utilized to calibrate the speed of the chain conveyor. The conveyor speed was set to different values, and the quality grade information of cucumbers, denoted as 1, 2, or 3, was directly transmitted to the PLC in the system. This process enabled the determination of the capacity of the grader at different speeds.

#### 3.1.2. Analysis of Experimental Results

**Figure 11:**
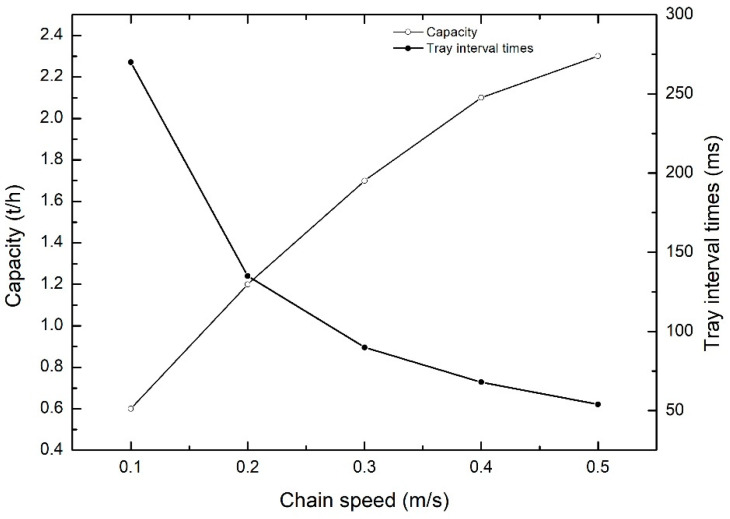
Capacity of the grader at different chain speeds.

Figure 11 illustrates the capacity of the grader at chain speeds ranging from 0.1 m/s to 0.5 m/s, achieving a capacity range of 0.6 t/h to 2.3 t/h. Under chain speeds from 0 to 0.3 m/s, the capacity shows a linear increase with speed. The entire system operates stably, with long tray intervals and accurate readings from the photoelectric sensor for each tray. The grading actuating mechanism can make correct grading actions based on tray positions and bound quality information. Under chain speeds from 0.3 to 0.5 m/s, the capacity increase slows down. This is attributed to increased vibrations of the grader at higher chain speeds. Meanwhile, the short tray intervals lead to the photoelectric sensor mounted on the grader being influenced by equipment vibrations and interval times. This impact may result in missed readings or consecutive readings of vegetable trays, causing mismatches between tray position and quality information. Ultimately, this leads to the grading actuating mechanism failing to make the expected grading actions, reducing the overall capacity.

### 3.2. Comparison Experiments between Different Models

#### 3.2.1. Experiment Setup

We compared the performance of MassNet with AlexNet [34], MobileNet [35], and ResNet18 [36] on our collected cucumber mass dataset, with the hardware environment as shown in the Table 3.

AlexNet, MobileNet, VGG11, and ResNet18 are popular deep learning models in recent years, primarily used for tasks such as image recognition, classification, segmentation, etc. Few researchers have applied them to regression prediction tasks. In our experiments, we adjusted the output layers of these models to tailor them for regression tasks. These models, along with our designed MassNet, were individually trained on our collected cucumber mass dataset. Finally, the performance of each model was evaluated on the same test set comprising 100 samples.

To compare the performance between the actual mass and the prediction produced by regression based on different deep learning methods, the estimation metrics of root-mean-square error (RMSE) and mean absolute percentage error (MAPE) were calculated by Equations (2) and (3), respectively. The inference time is used to evaluate the real-time performance and response speed of different models.

(2) $$\mathrm{RMSE}=\frac{\sqrt{\sum (M_{i}^{\mathrm{predict}}-M_{i}^{\mathrm{actual}}{)}^{2}}}{N}$$

(3) $$\mathrm{MAPE}=\sum \left|\frac{M_{i}^{\mathrm{predict}}-M_{i}^{\mathrm{actual}}}{M_{i}^{\mathrm{actual}}}\right|\times \frac{100\%}{N}$$

#### 3.2.2. Analysis of Experimental Results

Table 4 shows the results of cucumber mass prediction for different models. According to the table, our designed MassNet achieves the best performance in predicting cucumber mass compared to other models: RMSE is 6.65 g, MAPE is 3.9%. AlexNet and MobileNet are primarily employed in large-scale image recognition and classification. During the feature extraction process, they utilize techniques like local normalization and depthwise separable convolutions. While these methods focus more on shallow and local features, they limit the ability to learn complex features, resulting in poorer predictions for cucumber mass. ResNet introduces residual connections, addressing issues like gradient vanishing and exploding during training. This helps stabilize gradient propagation, enabling the training of very deep neural networks. In predicting cucumber mass, ResNet outperforms AlexNet and MobileNet, but it comes with significantly longer inference times.

Our designed MassNet, built upon residual connections, further incorporates cross-stage partial connections. This is achieved by alternating convolutional layers and CSPC layers, extracting higher-level abstract features while reducing the number of layers and consequently decreasing inference time. MassNet achieves a well-balanced performance in both prediction accuracy and inference efficiency.

### 3.3. Online Grading Experiment

#### 3.3.1. Experiment Setup

A total of 100 fresh cucumbers of the same variety were selected, and their masses were recorded using an electronic scale. The quantities for each grade (1, 2, 3) were 34, 15, and 51, respectively. The weighed cucumbers were placed sequentially on the vegetable trays of the grading machine running at 0.1 m/s. The predicted mass for each cucumber and the overall grading efficiency of the machine were obtained. Formula (4) for grading efficiency was determined by P. Rajkumar [23].

(4) $$\eta_{0}=\frac{T_{N}-T_{M}}{T_{N}}$$

where $\eta_{o}$ is the overall grading efficiency in %, *T_N_* is the total number of cucumbers obtained from all grades, and *T_M_* is the total number of misgraded cucumbers obtained from all grades.

#### 3.3.2. Analysis of Experimental Results

**Figure 12:**
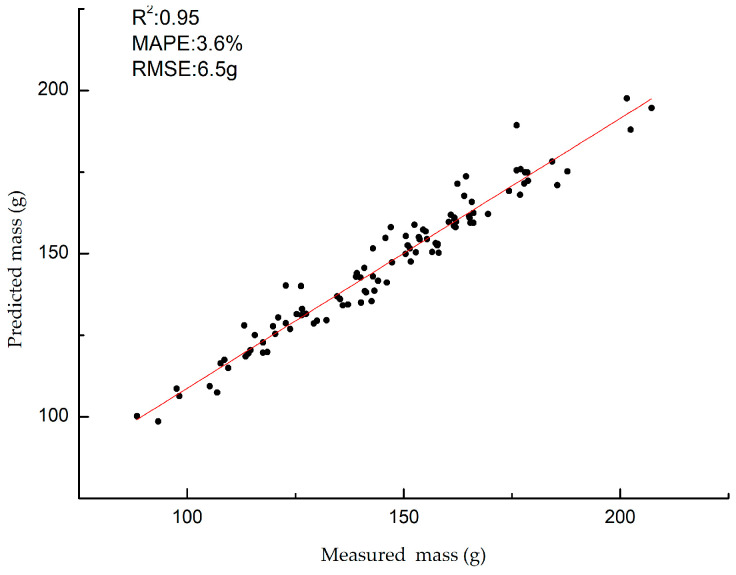
The relationship between the actual measured values and predicted values of the mass.

Figure 12 illustrates the relationship between the actual measured values and predicted values of the mass for 100 cucumbers obtained through the vision algorithm in the online grading experiment. The graph shows a strong linear correlation between the actual and predicted cucumber mass values, with an R^2^ of 0.95, MAPE of 3.6%, RMSE of 6.5 g, and grading speed of 60 pcs/min.

Table 5 presents the grading efficiency of the cucumber quality grader. In the online grading experiment with 100 cucumbers, a total of 7 cucumbers were misclassified (e.g., grade 1 misclassified as grade 3, or grade 2 misclassified as grade 1), resulting in a grading efficiency of 93%. At a chain speed of 0.1 m/s, the grading speed of the quality grader is 60 pcs/min, which is six times faster than manual grading. Importantly, no cucumbers suffered damage throughout the entire grading process.

The designed MassNet model, trained on a relatively small dataset of cucumber mass, demonstrated excellent performance in practical online grading tests for the following reasons. Firstly, in terms of cucumber characteristics, under the same agricultural management and growth environment, cucumbers exhibit similar appearance traits (length, diameter, curvature) within a certain range. Therefore, collecting a small-scale dataset in the same cultivation area may cover most cucumbers with different appearance characteristics. Secondly, on the algorithmic side, when establishing the dataset, cucumbers were placed on trays to ensure consistency between the image background and the cucumber quality grader. The simple background allows the neural network to focus more on the cucumbers. Furthermore, the designed MassNet network alternates between convolutional layers and CSPC layers, extracting more high-level feature information in concentrated cucumber image views to improve mass prediction accuracy. Thirdly, in terms of electromechanics, setting the conveying speed to 0.1 m/s ensures the relative stability of the tray carrying cucumbers during movement, guaranteeing clear capture of cucumber images. The low conveying speed provides ample image processing time and sufficient sensor response time; the output cucumber grading information can be accurately executed in coordination with the electromechanical system.

Compared to TriHuynh’s [28] use of traditional image processing methods to estimate cucumber quality based on the symmetry of cylindrical vegetables, MassNet shows better compatibility with asymmetric cylindrical vegetables. Weijun Xie [37], in their study on 1000 carrot images, extracted six dominant features of carrots and predicted their quality using a combination of four machine learning methods. In comparison to their approach, MassNet performance in cucumber quality prediction is slightly inferior, partly due to the differences in characteristics between carrots and cucumbers, as our collected cucumbers include flowers. Additionally, the smaller scale of the cucumber dataset contributes to this difference. However, MassNet effectively utilizes pixel information to extract implicit features, achieving satisfactory results even with a small dataset. Furthermore, we applied the obtained mass prediction results directly to the grader, demonstrating the feasibility of online grading. The cucumber grading process benefits from the protection of vegetable trays, unlike Yee-Siang Gan’s [15] roll-based cucumber grading system. In the designed quality grader, the grading mechanism operates on vegetable trays, ensuring that cucumbers remain undamaged during the grading process.

## 4. Conclusions and Future Work

The cucumber quality grader designed in this paper incorporates a novel grading mechanism—the fixed tray type—ensuring that cucumbers are not damaged during the grading process in the mechanical design. In the electrical design, a real-time synchronization and tracking strategy for vegetable quality information and position is employed, enabling trays carrying cucumbers to achieve grading during motion and ensuring the equipment’s capacity. Experimental results indicate that, at speeds ranging from 0.1 to 0.5 m/s, the designed cucumber quality grader achieves a capacity between 0.6 and 2.3 t/h. In the visual algorithm, a MassNet network based on CNN is designed, which eliminates the need for extracting complex explicit features from cucumbers. Mass prediction for cucumbers is achieved solely through an RGB top view. Compared to AlexNet, MobileNet, and ResNet, MassNet performs the best in predicting cucumber mass: RMSE is 6.65 g, MAPE is 3.9%. In online grading experiments, 100 cucumbers were successfully graded for mass, achieving a grading efficiency of 93% and a grading speed of 60 pcs/min.

In future work, on the algorithmic side, we will employ deep learning to explore the grading of other indicators for cucumbers, such as length, curvature, and surface damage, aiming to enhance the applicability of the cucumber quality grader. For mass prediction, accuracy can be improved by incorporating multiple perspectives and modalities, providing additional information for feature extraction. We plan to expand the cucumber dataset to increase the robustness and accuracy of the MassNet model for mass prediction. This expansion will involve introducing more variables related to rod-shaped vegetables (e.g., carrots, sweet potatoes) to augment the model’s generality.

In terms of grader optimization, efforts will focus on mitigating grader vibrations, optimizing sensors, and addressing issues related to the misalignment of tray positions and quality information under high speeds. These optimizations aim to improve the machine’s capacity. We also aim to design a loading and unloading device that seamlessly integrates with the quality grader, achieving full automation throughout the grading process.

## Figures and Tables

**Table 1:** Mass grading requirements.

Grade Indicator	Grade 1	Grade 2	Grade 3
Single cucumber mass (g)	≥150 ≤170	>170 ≤250	<150 >250

**Table 2:** Hyperparameter settings.

Parameter	Value
Optimization algorithm	Adam
Initial learning rate	0.0001
Epoch	100
Batch size	4

**Table 3:** Experimental environment hardware.

Computer Configuration	Specific Parameters
CPU	Inter E5
GPU	NVIDIA GTX4090
Operating System	Windows 10-x64
Random Access Memory	DDR4 64G
CUDA	11.8

**Table 4:** The results of cucumber mass prediction for different models.

Network	RMSE	MAPE	Inference Time
AlexNet	25.2 g	16.2%	20 ms
VGG11	23.9 g	15.3%	30 ms
MobileNet	12.3 g	7.1%	70 ms
ResNet18	10.0 g	5.8%	900 ms
MassNet	6.7 g	3.9%	280 ms

**Table 5:** Cucumber quality grader efficiency.

Grade Level	Test Number	Miss Grade Number	Grader Efficiency
1	34	1	93%
2	15	3	
3	51	3	

## Data Availability

The data presented in this study are available on request from the corresponding author. The data are not publicly available since future studies are related to current data.
